# The GP’s perceived role and use of language concerning the existential dimension of palliative patients: a Dutch interview study

**DOI:** 10.1186/s12875-022-01789-6

**Published:** 2022-07-26

**Authors:** Sara T. Busser, Jeanne Rens, Bregje Thoonsen, Yvonne Engels, Anne B. Wichmann

**Affiliations:** 1 Radboud university medical center, Nijmegen, the Netherlands; 2 Zuyderland Medical Center, Heerlen, the Netherlands; 3 GVPZ, an organization of spiritual care counsellors providing spiritual care at home, Nijmegen, the Netherlands

**Keywords:** General practitioners, Palliative care, Existential dimension, Spirituality qualitative research

## Abstract

**Background:**

Palliative patients have to cope with their disease and impending death. Knowing what this means for a patient is crucial for person-centred care. Although guidelines state it is a GP core task to explore existential issues of palliative patients, this is not standard practice.

**Aim:**

Exploring Dutch GPs’ perceived role regarding addressing the existential dimension of palliative patients, and which vocabulary GPs use when doing this.

**Design and setting:**

Qualitative study amongst Dutch GPs. Participants were recruited by purposive sampling and snowballing, considering gender, working experience and ideological personal beliefs.

**Method:**

Semi-structured in-depth interviews were performed, transcribed and analysed using content analysis.

**Results:**

Seventeen GPs participated. Three themes were identified: *Language*, *Perceived role* and *Practice*. Interviewees generally saw it as their role to pay attention to the existential dimension of palliative patients. However, not all knew how to define this role, or how to refer patients with existential struggles to a spiritual counsellor. The multidisciplinary Dutch guideline ‘Existential and Spiritual Aspects of Palliative Care’ seemed largely unknown. Interviewees mostly fulfilled their role in an intuitive, pragmatic way. Questions such as “*What does it mean for you to be seriously ill?*” or “*Do you have support from someone or something?*” fitted daily practice.

**Conclusion:**

This study emphasizes the importance of basic GP education in exploring existential issues. The coexistence of a professionally obliged attention and an intuitive approach seems to be in conflict. We recommend appropriate training for GPs, research on the potential enhancement of collaboration between GPs and spiritual counsellors and implementation of the relevant guideline on well-known platforms.

**Supplementary Information:**

The online version contains supplementary material available at 10.1186/s12875-022-01789-6.

## Background

Imagine death is on your doorstep. Having to say farewell to your loved ones, and at the same time having to deal with severe illness and its consequences. Being confronted with this reality often gives rise to life questions, and subsequent struggles are frequently reported amongst palliative patients [1]. Therefore, in palliative care, addressing the four interacting dimensions – physical, psychological, social and existential/spiritual – is internationally recognized as important, and even as a professional and ethical responsibility [2, 3]. It is emphasized that appropriate weight should be given to each dimension [3], and that existential/spiritual issues should be recognized, explored and integrated in care provision to palliative patients by every involved health care professional.

In recent years, initiatives have been established to promote the integration of this dimension of palliative care. In 2010, an EAPC Taskforce was founded which aims to foster evidence-based care, to improve staff competence and confidence, and outcomes for patients and carers on this dimension [4]. In 2018, the Dutch guideline ‘Existential and Spiritual Aspects of Palliative Care’ was released, providing health care professionals engaged in providing palliative care with relevant knowledge and tools [5]. This guideline is of interest European-wide, and is (being) translated in English, Italian, Danish, Finnish and a patient summary. In 2020, an EAPC white paper on multi-disciplinary education for this dimension in palliative care was published [6]. Progress is also evident at national levels.

GPs play a crucial role in care delivery for this patient group, as in the Netherlands they are responsible for arranging and delivering palliative care at home [7]. In the national GP training program it is stated as a competence that ‘A GP in training should be able to have a conversation about the approaching end of life and take into account existential issues or existential suffering’ [8]. Internationally, the WONCA describes that one of the GP core competencies is applying a holistic modelling on physical, psychosocial, cultural and existential needs in communication with patients and clinical tasks [3]. The need for multidimensional attention is also described in the above-mentioned guideline which has been approved by the Dutch College of General Practitioners [5, 9, 10].

In sum: international training plans and guidelines expect GPs to integrate the existential dimension in palliative care. Doing this might contribute to quality of end of life [11–15], and patients appreciate it [16, 17]. However, still little attention is paid to this dimension [10, 18–21]. Moreover, GPs less often collaborate with experts on this dimension, such as spiritual counsellors, than with other health care professionals [10, 22]. Most find integrating the existential dimension in palliative care important, but are unsure about how to properly implement it [23]. Lack of time, feeling incapable, having the idea it is not their role, seeing it as taboo or merely being related to religion are also found to be barriers [24–28]. Furthermore, finding appropriate words when talking about this dimension is shown to be difficult [10, 23].

Vermandere et al. in an evidence synthesis concluded that further research is needed to clarify the role of the GP, and found that GPs sometimes struggle with finding words to address this dimension [23]. Recently, a Danish and a Scottish study provided insights in how GPs in these countries perceive their role, and understand and apply the dimension [10, 29]. They found that GPs mainly address the existential dimension using their ‘gut feeling’ and recommended more explicit attention to existential issues of patients. Bornet et al. recently suggested developing and implementing a model helping GPs integrate it [30]. However, little is known about the GPs’ stance towards such models or guidelines, and about their perception of roles and responsibilities in addressing the existential dimension. Therefore, the aim of this study was to explore how Dutch GPs view and fulfil their role in addressing the existential dimension of palliative patients, and which vocabulary they use for this. Which words fit their practice?

As GPs within a secular European setting, like the Netherlands, are primarily concerned with secular existential orientations – such as meaning, value of life and personal values that are not centered in spiritual or religious ideological personal beliefs [9, 10] – this study employs the definition “existential” when it concerns this dimension. This is in line with the terminology of the WONCA, the academic and scientific society for general practitioners in Europe [3]. It is understood as the way persons experience, express and seek meaning and purpose in life [5]. However, as “spiritual counsellor” is the common definition used for experts on this dimension, we will apply this term when referring to this profession.

## Methods

### Research design

As experiences, opinions and thoughts are best explored using in-depth interviews, a qualitative research methodology with an interpretative approach was used [31]. In order to get as close to GPs’ actual communication as possible, interviewees were asked which questions they ask the palliative patients they provide care to, what themes they discuss and which words they use when talking about the existential dimension.

### Study population

Dutch GPs with experience in palliative care were included. Purposive sampling was done in order to reach variation, considering gender, working experience and ideological personal beliefs [32].

As participants were not subject to treatment, nor were they required to behave in a particular way, the Medical Review Ethics Committee concluded this study was not subject to the Medical Research Involving Human Subjects Act (file number: 20206577) [33].

### Data collection

#### Recruitment of participants

Between June and August 2020, GPs working in the South-East of the Netherlands from the professional network of the researchers were approached by telephone or e-mail. After each interview, the interviewee was asked if he or she knew another GP who could be invited, preferably with different ideological personal beliefs (snowballing) [34]. Recruitment was continued until data saturation occurred, after which a couple more confirmative interviews were done [35, 36].

#### The interview

The interviews were conducted by SB with the use of an interview guide (Supplementary Table S1) which was created based on a literature study and two exploratory meetings, one with a Palliative Home Care Group (PATZ) and one with a GP specialized in palliative care. See Table 1 in main text for researcher characteristics. At the start of the interview, palliative patients were defined as patients about whom the participating GP would not be surprised if they would die within a year: the so called ‘Surprise Question’ [35, 36].

**Table 1:** Characteristics researchers

Initials	Relevant characteristics
SB	Master medical student, Radboudumc university medical center, the Netherlands.
JR	Spiritual counsellor, Zuyderland medical center and GVPZ, the Netherlands.
BT	General practitioner, Nijmegen, The Netherlands.
YE	Professor in ‘Meaningful Healthcare’, Radboudumc, the Netherlands. Expert in palliative care and integrating contextual issues in patient - healthcare professional communication.
AW	Postdoctoral researcher in palliative care and contextual care, Radboudumc, the Netherlands.

In order to distinguish the role of GPs from the role of experts in care regarding the existential dimension, the advised ‘ABC’ scheme from the Dutch guideline ‘Existential and Spiritual Aspects of Palliative Care’ was applied. See appendix for a summary of this guideline [5]. (A) refers to “Attention”, (B) to “Accompaniment”, *Begeleiding*, in Dutch and C to “Crisis and referral”. This scheme states that each health care professional involved in the care for palliative patients, should have attention for the existential dimension, accompany patients if desired, and refer when a crisis is identified.

All interviewees gave a priori verbal consent to audio-record the interview and to anonymously use the data. Most took place face-to-face (at a distance of > 1.5 m due to COVID-19 regulations) at the interviewee’s general practice. Some interviews were conducted by telephone or by video calling, based on the preference of the interviewee.

The interviews were transcribed verbatim, identifiable information was removed. Afterwards, transcripts were sent to interviewees as member check. If necessary, the interviewer asked to clarify certain aspects of the transcript, or to further explain and/or check the interpretation.

### Data analysis

Transcripts were read and reread by SB and AW before starting the analysis. An inductive approach was adopted to allow openness for issues coming up during the interviews. By means of thematic conventional content analysis [37], codes emerged from the data. Analysis took place iteratively: interviews and analysis were alternated. Atlas.ti software version 8.4.20 was used.

The first four interviews were coded independently by SB and AW and discussed in order to reach consensus. The following were coded by SB, and discussed with AW if needed in order to limit subjectivity. In a peer group session with SB, AW and YE, codes were categorized and subsequently thematised using a code tree. See Supplementary Table S2 In presenting this study’s findings, the 21-item Standards for Reporting Qualitative Research (SRQR) guidance was used [38].

## Results

31 GPs were approached. Participation rate was 17/31 (51%). Reasons for non-participation were lack of time, no response, no interest. Seventeen in-depth interviews with a length of 45-60 minutes were conducted between June and September 2020. See Table 2 in main text for information regarding interviewees. Data saturation was reached after fifteen interviews. Three themes (‘Language’, ‘Perceived role’ and ‘Practice’) were identified in the 66 codes and 13 categories.

**Table 2:** Characteristics interviewees

	Gender	Age	Years experience	Estimated number of palliative patients taken care of (in total)	Ideological personal beliefs GP	Practice	Patient Characteristics
1.	F	55	25	75-100	Humanist	Healthcare centre in Nijmegen	• Slightly younger than average population. • Group Vietnamese/Moroccan. Non-religious/Catholics/Muslims.
2.	F	56	25	> 100	Raised Roman-Catholic, now atheist	Practice (2 GPs) in Ravenstein	• 22% above age of 60. • Mostly catholic, a few Islamic.
3.	F	64	32	100	Protestant	Practice (2 GPs) in Oss	• Mainly between 40 and 60 years. • Originating from more than 30 countries, variating Christians (Catholic, Protestant, Russian-Orthodox), Muslims (Orthodox and Alevites), Hindustani, non-religious. Many patients with a low socio-economic status, substantial part low literate.
4.	F	35	4	14	Humanism and rationalism	Healthcare centre in Nijmegen	• Mainly Christian (practising/not practising) and atheist, small group Muslims, also an even smaller Asian population (Vietnamese and Chinese) who are Buddhist.
5.	F	36	6	30	Atheist	Healthcare centre in Nijmegen	• Relatively few elderly people in total practice, in my own a little more. • Lots of different cultural backgrounds. Quite a lot atheist, sometimes Muslims, sometimes Christian. Many Hindu people.
6.	M	45	14	55	Raised reformed protestant, now agnostic	Healthcare centre in Nijmegen	• Younger than average population. • From Buddhists to Catholics, from Muslims to atheists. Many different nationalities, 25% not of Dutch origin. Low socio-economic status.
7.	F	42	10	65	Agnostic	Healthcare centre in Nijmegen	• Average younger population. • Cultural background unknown.
8.	F	34	3	5	Atheist	Locum GP in Nijmegen	• Mixed population. • Relatively more people with low socio-economic status, Native Dutch 60% immigrant 40%. Muslims 20, 10% active Christian 10%, remaining percentage atheist.
9.	F	47	18	5	Traditionally Catholic, not practicing	Healthcare centre in Nijmegen	• Average younger population. • Around 30% immigrants, many different cultural background. Low socio-economic status.
10.	M	33	3	30	Muslim	Locum GP in villages and city and GP in Asylum Seekers Centre	• Different populations, both young/old. • Both religious and non-religious. Very diverse.
11.	M	61	29	500	Catholic background, now humanist	Practice (3 GPs) in Udenhout and GP in institution for persons with a mental disability	• Relatively many elderly people in practice. • Village practice in a Catholic community. Some Moroccan background (about 40-50 people). For the rest, mainly native Brabant.
12.	M	**52**	20	250	Familiar with Catholic themes and rituals, but no longer finding strength in religion	Practice in Oss	• Average practice, slightly more elderly. • Multicultural neighborhood. From Catholic (70%) to several hundred Protestants. Muslims (10-15%), but also Buddhism (150 Asians in practice).
13.	M	37	2	10	Atheist	Healthcare centre in Nijmegen	• Average young population. • Many different cultural backgrounds, many psychosocial problems.
14.	M	61	28	100	Christian raised and practicing	Practice (3 GPs) in Hoevelaken	• Former Christian community, current population is 60% imported, often highly educated commuters who work in Utrecht/Amsterdam, with a pragmatic agnostic philosophy of life.
15.	M	43	15	40	Roman-Catholic	Healthcare centre in Oosterhout	• Mostly young highly educated population.
16.	F	41	8	50	Muslim	Healthcare centre in Arnhem-Zuid	• Even mix of young and old. • Diverse population; many with a Muslim background, but also non-religious, Protestant/Catholic. Many patients with a low socio-economic status.
17.	F	43	10	25	Protestant	Solo practice in Veenendaal	• Urbanized countryside. More elderly. • Variety in backgrounds: Muslim, Protestant, mixed. Partly immigrant population. Relatively low socio-economic status.

### Theme 1. Language

#### Definition

The initial question in the interviews was: what are your first thoughts and ideas regarding the existential dimension, what does it provoke? All interviewees saw it as a broad dimension, certainly not only about religion. *“The existential dimension is very broad. I associate it with what people find important in their lives, what they get support from, what or who they turn to when they experience very hard things.”* (R3) However, most found it difficult to define. It provoked multiple associations, including negative ones. *“I always think it is very dusty, a bit old-fashioned, musty, even though I am not the youngest myself (...) I think I would not use that word that quickly.”* (R17).

In addition, interviewees were asked what it means in the care for palliative patients. According to several interviewees, it is about how patients find inner peace and give purpose to life and dying. *“You want people to find good closure of their life.”* (R15).

#### Vocabulary

Interviewees mentioned they discuss a wide variety of issues regarding the dimension with palliative patients, e.g. relationships, family, hobbies, nature, a pet, deeper wishes, religion, concerns, problems, grief, guilt, finding closure, wanting to die at home.

Many interviewees noted that the existential dimension is intertwined with the physical, social and psychological. Comparable examples showing this interconnectedness (e.g. regarding pain) were mentioned a number of times. *“I think it is very good to not only approach pain from a medical technical point of view by examining someone physically. Part of the pain treatment is also about exploring the existential issue: ‘is your body telling you something greater than the metastasis in your bone? ...or do you find that pain hard to bear because you are afraid of dying and how come so?’”*(R6).

Sometimes, during the course of the interview, interviewees became aware of the fact (s)he actually did recognize existential questions, but did not classify them as such. They until that moment during the interview had not realized the earlier mentioned interconnectedness.

Interviewees adapted their vocabulary to connect with the patient. Questions the interviewees asked palliative patients to explore the existential dimension were for example: “*What are last wishes you have?*” (R13); “*How do you envisage your dying process, and why do you see it that way?*” (R13); “*What does it mean for you to be seriously ill?*” (R2); “*Do you have support from someone or something? Thoughts? Religion?*” (R1); “*What is important to you, to your family, your husband? (…) Can you share your sorrow?*” (R4).

### Theme 2. Perceived role

#### Attention

Generally, interviewees saw attention to existential issues of palliative patients as important, and their role to explore and pay attention to it. *“Based on my belief that an important part of being able to die in a good way, is the question: ‘Can I look back at my life with a good feeling?,’ I will always explore, mention and discuss the existential dimension.”* (R6) One firmly stated every GP should pay attention to this dimension. If this interviewee sees a palliative patient during out of hours care and nothing is documented about important wishes, he calls him or her the following day. Because, *“That is literally the question I ask my colleagues: ‘If it were your parents, if it was your partner, how would you like that? Do you want seven different doctors at your bedside over a weekend?’ “No, no.“ Well, then do something about it.” (*R15).

Not all GPs were certain about their role. In response to the guideline’s statement that every health care professional should pay attention to the existential dimension of palliative patients [5], one interviewee critically remarked *“Why? You can still be a good doctor if you do not master this aspect. (…) I think ‘should’ is a very big word. In the sense that, if you can’t, you cannot provide good palliative care.”* (R12) However, the same interviewee later on stated that: *“I would think it is a bad GP if he or she pays no attention to it at all. (…) Then, as a GP, you really should scratch your head like: “what am I doing anyway?”* (R12).

One interviewee even considered the GPs’ role to be less important. *“I don’t see a very big role here for the GP. I always think that people, especially adults, think about this for themselves.”* (R8).

#### Accompaniment

Part of the interviewed GPs also saw accompaniment as the responsibility of the GP. Some interviewees called themselves ‘coach or catalysator’ to symbolize their role in stimulating patients to think about existential questions. *“Because it concerns every person... the most blunt or stiff patient still has life questions and fears… and from the relationship and the history you have built as a GP, I think you are the appropriate person to open-up that part...”* (R14).

#### Crisis

In some cases, the GP needs support in care for the existential dimension or wants to refer the patient to an expert. Sometimes interviewees referred a patient to a psychologist, practice nurse mental care (POH-GGZ) or pastor. Around half of the interviewees also offered the option of referral to a spiritual counsellor. Several reasons were mentioned for not referring. Some felt the trust relationship built with a patient, makes it hard to introduce a new person. Other reasons were not having considered the option, or not being able to find their way. *“People are more familiar with social work, the general practitioner, the POH-GGZ. (...) I would not immediately know whether a spiritual counsellor is part of our palliative team. In the hospital: yes. But in primary care? I don’t know.”* (R3) Opposite to this, another interviewee, working in the same region, explained *“There is a webpage for that! You go there* (clicking on website, red.) *network palliative care…Yes, refer to a spiritual counsellor. That is what I sometimes do after a few conversations.”* (R12).

#### Competence

Many interviewees voiced they had a natural interest in the context of the patient. According to some, discussing the existential dimension falls under the scope of *“generalist thinking”* (R13) or *“context medicine”* (R11) and GPs therefore are capable and adequately trained to integrate it. Others however had doubts about their competence and did not trust their own capability in discussing existential issues. This mainly was the case when GPs had lesser experience in care for palliative patients. A part indicated it is unclear what the dimension exactly entails, and what is expected of GPs. *“If you are competent, then you also need to know what you are talking about. I don’t know if I really cover the entire field for 100 percent concerning the existential dimension..”* (R17).

#### Meaningfulness GP

Although not all interviewees felt competent, most experienced conversations regarding this dimension a very valuable part of their profession. *“It gives a very satisfying feeling when you turn out to be of significance to someone. (…) It even moves me.”* (R3).

### Theme 3. Practice

#### How do GPs integrate the existential dimension?

What emerged from all interviews: the existential dimension is explored in an intuitive way. There’s no standard approach, no checklist or standard question. *“You always just make up on the spot how you talk and what you do. Of course I don’t have a list.”* (R3) The dimension is dealt with in conversations along the way and depending on personal circumstances. *“You cannot completely frame it ... there are questions that I always ask, but never very consciously. More gradually I think: ‘Oh yes, I still want to know this in order to be able to provide good care.’”* (R2).

In some interviews, GPs only during the course of the interview realized some things they discuss in their practice actually concern the existential dimension. For example, one interviewee did not realize a patients’ wish for euthanasia could be based on existential suffering, and only became aware of this during the interview. *“I have had a euthanasia request from a patient who, from a medical point of view, did not hopelessly and unbearably suffer. She just didn’t want to live anymore, and had some pain complaints ... The SCEN doctor (independent specially educated doctor who should always be consulted in euthanasia cases in the Netherlands) argued that if she had multiple problems and this wish, it should be allowed. But this really did not fit for me, at all.. However, I again went to ask the patient to what extent she possibly also felt sort of depressed... and then she affirmed... So I might have another conversation with her about the existential dimension ... because I haven’t done this explicitly yet, and I am actually quite curious about what will come from that.”* (R9).

#### A pragmatic approach

Most interviewees had a pragmatic attitude towards the existential dimension in palliative care. Subjects discussed with patients often focused on what can still be arranged or done. *“I am focused on practical solutions and am goal-oriented.”* (R4).

#### Conditions

Interviewees noted that addressing the existential dimension requires an open attitude, time and ‘being unhurried’. For example, during home visits at the end of the day. Some stated that they did not have enough time. Moreover, a trusted relationship is important.

#### Guideline and need for training

A large part of interviewees was not familiar with the guideline ‘Existential and Spiritual aspects of Palliative Care’, [5] or “had seen it once” (R1, R17). One interviewee however, knew the guideline well and used it to find inspiration for questions to explore the existential dimension: *“When we are in the palliative phase I can think: ‘Gosh, there are a lot of existential issues here I need to explore...’ then I sometimes open the guideline…'what questions were there ... oh yes’, then I already remember, and get inspired.”* (R7).

Uncertainty of GPs was found regarding whether it specifically has to be the GP paying attention to this dimension. Autonomy seemed to be an important core value of the interviewees. And when it came to ‘should’, ‘competence’ or ‘guidelines’, this often elicited reactions like *“I think the more you frame the existential dimension... and start thinking what competence do you need for that ... the more dangerous it becomes, that it will happen according to fixed rules and that fixed questions have to be asked, and if you have not asked that question, then you have not passed.”* (R11) However, another more experienced interviewee seemed to be suggesting some form of assessment: *“I don’t know if a GP is competent for that. Every GP will have to look at this for himself and I think he can be assessed on that. I believe there is a guideline... but I don’t know this guideline, well .. there you have it! (laughs) Maybe it contains very interesting things that I don’t apply at all and maybe I’m completely incompetent.”* (R6).

## Discussion

### Summary

This study gives insight in how Dutch GPs in a largely secularized society view and fulfil their role in addressing the existential dimension of palliative patients, and which vocabulary they use for this. A wide variety of issues are brought up in practices of interviewed GPs (ranging from a pet, to deep wishes or grief) which are probed using questions like “*What does it mean for you to be seriously ill?*” or “*Do you have support from someone or something?* The dimension is seen as broad and difficult to capture. Generally, interviewees saw it as their role to integrate the dimension in their palliative care. However, it was not always clear to them what this role entails, nor did they all feel competent in adopting this role. For example, most did not know how to refer to an expert, e.g. a spiritual counsellor. Interviewed GPs fulfilled their role intuitively, with from time to time a pragmatic attitude.

### Strengths and limitations

Although the relevant multidisciplinary guideline gives guidance regarding the role of the GP in integrating the existential dimension in palliative care [5], this study provides new information on the *own* perceived role of a diverse group of GPs and on whether the content of the guideline actually fits their practice. It is the first European study that the authors know of to explore how GPs talk about this dimension and which vocabulary they use. The diversity in experience of the interviewed GPs enriched our data. However, it was harder to generate a general conclusion. A disadvantage is that statements about the actual communication of GPs regarding the existential dimension could not be made, as only answers to the interviewers’ questions were available. Actual GP-patient conversation recordings might have provided richer information.

### Comparison with existing literature

#### Theme 1. Language

A wide variety of issues concerning the existential dimension is being discussed in the practices of interviewed GPs, ranging from a pet to deep wishes or grief. Participating GPs therefore mostly asked ‘casual’ questions about what is of importance to the patient, such as “*What does it mean for you to be seriously ill?*” or “*Do you have support from someone or something?*” Those questions are in line with the Mount Vernon Cancer Network questions [39], which therefore seem to well-fit GP practice. They are: *How do you make sense of what is happening to you? What sources of strength do you look to when life is difficult? Would you find it helpful to talk to someone who could help you explore the issues of spirituality/faith?* Nevertheless, most interviewees felt the dimension is broad and difficult to capture in concrete language, which is in line with previous studies showing GPs’ struggles with the language surrounding this dimension [23].

Moreover, our study, just as others have [10, 40–42], showed the interconnectedness of the existential with other dimensions. However, this interconnectedness is not always recognized, which can lead to the existential dimension remaining underexposed. Life questions and subsequential struggles, which are frequently experienced amongst palliative patients [1], might therefore not be sufficiently addressed. Communication training and peer intervision could help GPs in recognizing and experiencing the interconnectedness of the existential dimension with the physical, social and psychological and improve multidimensional palliative care.

#### Theme 2. Perceived role

Generally, interviewees agreed attention for the existential dimension is important and meaningful. Most considered it their role, although some did not have a clear understanding of it, nor felt competent in it. Previous research showed similar results, and indicated it often is not clear to GPs what attention to the dimension entails, and that only few feel adequately trained [43]. However, guidelines expect attention to the existential dimension of palliative patients from GPs [2, 24]. A GP should be able to refer to or collaborate with a spiritual counsellor when existential questions are more complicated and more existential care is needed. Further research should find ways to help GPs get a clearer understanding of what their role entails and to empower them to take this role.

#### Theme 3. Practice

This study found that when GPs pay attention to the existential dimension, this is mainly done in an intuitive way, without a standard approach. As a consequence, the dimension might be missed, also in important situations. This was confirmed in other research showing GPs primarily depend on their “gut feeling” in addressing the existential dimension [10]. Although this gut feeling has been found to be valuable [44], it is not clear to which extent it is subjective to personal biases and preconceptions.

Attention to the integration of this dimension by GPs is only discussed in elective education or optional courses, and competence is not assessed. Although in our study standardization did not seem to be desired, others promote more professionalization in order to make care for this dimension more transparent [45]. Appleby et al. concluded their review by stating that if agreed it is desirable to encourage the integration of this dimension in general practice, we have to find common frameworks for these discussions [46], and a recent study amongst Scottish GPs even suggested to define a gold standard [47]. Wenham et al. in a review proposed core content, aims and objectives to guide further work on this [48]. We recommend research on ways of warranting integration of this dimension in care for palliative patients, without disregarding the GP as a professional in a personalised approach. For example by exploring what kind of training suits GPs’ needs and how attention for the existential dimension can be implemented by all GPs without a standard approach. Secondly, the desirability of competence assessment needs further investigation.

The relevant Dutch multidisciplinary guideline seemed not well known, nor implemented. Even though the Dutch College of General Practitioners (NHG) approved the guideline, it is not shared on their platform [49], and ‘unknown makes unloved’. Moreover, our study shows that some GPs better find their way to expert counsellors for referral than others. This did not seem to be influenced by region or level of experience. A reason not to refer was simply not having considered the option. Earlier studies found the same [10, 22, 50]. It is recommended to publish the guideline on the relevant platforms and further specific research should find out why some GPs collaborate easier with spiritual counsellors than others. To bridge this gap, we suggest for example to inform GPs better on how to refer to a spiritual counsellor, its possible benefits [21], and the fact that Dutch GPs since 2019 can refer to such a counsellor for free [51]. Or by cooperating with spiritual caregivers in multidisciplinary meetings, which has been shown to strengthen care in the existential dimension [52].

Lastly, this study showed a pragmatic attitude towards the integration of the existential dimension in palliative care by GPs. Earlier studies also found ‘high levels of pragmatism’ amongst GPs [29, 46]. In what way does this attitude of GPs influence if and how issues regarding this dimension are discussed?

### Implications for research and practice

Not all GPs feel competent in addressing the existential dimension of palliative patients, nor know how to refer to a specialist in the field. We recommend communication training and peer intervision for GPs in recognizing and experiencing the interconnectedness of the existential dimension with the physical, social and psychological. Moreover, GPs often address the existential dimension of the palliative patients they provide care to in an intuitive, pragmatic way. Warranting integration of this dimension, without disregarding the GP as a professional, is needed. It therefore is of importance to integrate basic education on addressing the existential dimension in the GPs training program. In addition, research into the desirability of competence assessment is recommended. Lastly, the Dutch guideline ‘Existential and Spiritual Aspects of Palliative Care’ should become available on relevant GP platforms.

## Conclusions

Although GPs have an important role in paying attention to the existential dimension of palliative patients, barriers exist. Besides, little was known about how GPs currently integrate this dimension in their care. Our study gives insight in Dutch GPs’ view on their role in integrating this dimension in the care for palliative patients, and how it fits their daily practice. It discovered a conflict between professionally obliged attention and the intuitive approach which GPs seem to use. We recommend appropriate training for GPs, more research on the potential enhancement of collaboration between GPs and spiritual counsellors and implementation of the relevant guideline on well-known platforms.

## Supplementary Information

**Additional file 1.** Interview guide

**Additional file 2.** Code tree

## Data Availability

Data are available upon reasonable request at the corresponding author.

## Abbreviations

GPs: General practitioners
NHG: Dutch College of General Practitioners
Patz: Palliative Home Care Group
POH-GGZ: Practice nurse mental care
SCEN doctor: Doctor for Support and Consultation in Euthanasia in the Netherlands
SRQR: Standards for Reporting Qualitative Research
