# Supplementary material for: The GP’s perceived role and use of language concerning the existential dimension of palliative patients: a Dutch interview study
Source: BMC Prim Care. 2022 Jul 26;23:182. doi: 10.1186/s12875-022-01789-6 (PMC9315078; doi:10.1186/s12875-022-01789-6)
Supplement: Supplementary file 1 — Additional file 1. Interview guide [file 12875_2022_1789_MOESM1_ESM.docx]

INTERVIEW GUIDE

Semi-structured in-depth interview

- What this research is about

Research question: how do GPs in the Netherlands perceive their role with regard to attending to the existential dimension of palliative patients, and what words do they use when talking about this topic?

Palliative patients: patients you would not be surprise to see die in the coming year (Surprise Question)

According to the WHO definition, palliative care involves four dimensions (psychical, psychological, social and existential). According to the interdisciplinary Dutch guideline “Existential and spiritual issues in the Palliative Phase, every health care professional involved in care for palliative patients should have attention for the existential dimension as well.

| ***Are you familiar with this guideline?***  *🡪 Probing to get to know how general practitioners (GPs) think about and deal with this guideline.* |
| --- |

There are main topics during this interview, and there are some sample questions which can be used to ask more questions (probes). Underlined text is background information for interviewer.

| ***I would like to know how you see your role in your practice, and how you understand the word “meaning” / the “existential dimension”?***  *🡪 Probes: What do you think this dimension of palliative could or should include? What does the word or dimension evoke?*  Consciously broad question: it needs to be clarified how GP sees the meaning of life. Freedom to talk about personal ides as well. Underlying question: do doctors with a different cultural background/particular belief system deal differently with the existential dimension in their care for palliative patients? The quantity and quality of education, cultural, philosophical or personal experiences of GPs may be related to how they view their role in this topic. This does not need to be specifically asked or explored, but when brought up by the GP, may be further explored.   - *How do you see your role, as a GP, in relation to paying attention to the existential dimension of your palliative patients?* - *How do you view the “assumption” in the guideline that this should be part of everyone’s palliative care work?*   Health care professionals identify and pay attention to existential issues. In case of crisis, referral should be made. If barriers (e.g. lack of time) are mentioned, interviewer may respond by asking how this might affect treatment, decisions, doctor-patient relationship and efficiency.  *🡪 Probes: what is (not) part of that role? Attention pro-active or reactive (when asked by the patient)? What makes a GP competent in paying attention to this dimension? Should GPs be competent? Do you feel competent? Why? If not: how do you deal with this? What kind of education have you had about palliative care? What ideas do you have for palliative care education? When would you use the guideline more quickly?* |
| --- |

| ***What topics/themes come up when you talk with palliative patients about the existential dimension?***  *Probes: which words/phrases are used? Do you ask questions, and if so, what are they? What kind of topics do patients come up with when they talk about this dimension? Do you have an example situation? How do you find a way to talk about this dimension? (How) does this differ from patient to patient? Are there certain moments/reasons to start a talk about this dimension? When do you see that someone is struggling? When is it particularly relevant to pay attention to this dimension? When do you do this (during visits or consultations)? Would you appreciate having tools available to help you with this? What tools do you use? What do you think about sample questions or tools to help you with this (e.g. Ars Moriendi, FICA, MVCN)?* |
| --- |

- Round-off

*Do you know GPs who have a different view or opinion to yours on this issue? Would you like to add anything that has not been mentioned yet? Do you have any questions? Contact details (for questions and additions afterwards) Would you like to receive the transcript, for adjustments or additions? Thanks*
