# Supplementary material for: The GP’s perceived role and use of language concerning the existential dimension of palliative patients: a Dutch interview study
Source: BMC Prim Care. 2022 Jul 26;23:182. doi: 10.1186/s12875-022-01789-6 (PMC9315078; doi:10.1186/s12875-022-01789-6)
Supplement: Supplementary file 2 — Additional file 2. Code tree [file 12875_2022_1789_MOESM2_ESM.docx]

| **Codes (66)** | **Categories (13)** | **Themes (3)** |
| --- | --- | --- |
| Questions to explore existential dimension  Themes and words conversations existential dimension  Interconnectedness with other dimensions  Support by faith | Vocabulary | Language |
| Definition of ‘meaning’ in existential dimension according to GP  Meaningfulness as giving purpose to life and dying by patients  Finding inner peace (dying)  Existential dimension relatives  ‘Meaning’ difficult concept  'Gut' feeling word ‘meaning’  Broad concept ‘meaning’  Meaningfulness for each patient (not only palliative) | Definition |  |
| Perceived role GP – exploring  Importance of acting proactively  Perceived role GP – attention/recognizing | Attention | Perceived role |
| Perceived role GP – accompaniment  More attention to other dimensions | Accompaniment |  |
| Perceived role GP – crisis  Reasons to refer  Reasons referral existential/spiritual counsellor  Reasons not to refer  Role other involved (within healthcare)  Role other involved (outside healthcare)  Involving minister/pastor/imam/rabbi  Involving relatives discussing dimension | Crisis and referral |  |
| GP does not feel competent  Competence discussing existential dimension (e.g. reflectivity) | Competence |  |
| Meaningfulness GP  Necessity of mutual trust to discuss existential dimension  Get to know your palliative patient, including existential dimension  Attention to existential dimension is part of context medicine  Influence personal ideas and convictions GP on discussing existential dimension  Influence life experience GP on discussing existential dimension | GP |  |
| How does GP integrate existential dimension?  Finding leads to explore existential dimension  Not being able to discuss existential dimension with patient  When GP finds it difficult  Focus patient on continuing with treatments  And if there is no meaningfulness left? | Integration | Practice |
| Open attitude GP  Being unhurried  Making time  GP does not have time | Conditions |  |
| Influence of intelligence patient on discussing existential dimension  Influence of culture patient on discussing existential dimension  Influence of age patient on discussing existential dimension  Influence of life story patient on existential dimension  Influence of relationship doctor-patient on discussing existential dimension  Connect to patient | Patient |  |
| Intuitive approach existential dimension  No checklist or standard approach  Existential dimension happens naturally  Process of discussing existential dimension  Narrow focus on physical dimension decreases in later phase  Palliative care past vs. present | Unawareness |  |
| Existential dimension is essential  Tools GP integrating existential dimension  Practical approach existential dimension  Pragmatic attitude GP on attention to existential dimension  Attitude GP toward religious issues | Conscious |  |
| Familiarity GP with guideline  Autonomy GP  Education or training on existential dimension in palliative care  Needs of GP concerning education in existential dimension  Experiential learning  Discuss with colleagues | Education |  |
